# The effects of probiotics, prebiotics, and synbiotics on polycystic ovarian syndrome: an overview of systematic reviews

**DOI:** 10.3389/fmed.2023.1141355

**Published:** 2023-06-09

**Authors:** Pooneh Angoorani, Hanieh-Sadat Ejtahed, Fateme Ettehad Marvasti, MinaSadat Taghavi, Behzad Mohammadpour Ahranjani, Shirin Hasani-Ranjbar, Bagher Larijani

**Affiliations:** ^1^Obesity and Eating Habits Research Center, Endocrinology and Metabolism Clinical Sciences Institute, Tehran University of Medical Sciences, Tehran, Iran; ^2^Microbiota Research Group, Endocrinology and Metabolism Research Center, Endocrinology and Metabolism Clinical Sciences Institute, Tehran University of Medical Sciences, Tehran, Iran; ^3^Department of Pediatric Cardiology, Bahrami Hospital, Tehran University of Medical Sciences, Tehran, Iran; ^4^Endocrinology and Metabolism Research Center, Endocrinology and Metabolism Clinical Sciences Institute, Tehran University of Medical Sciences, Tehran, Iran

**Keywords:** polycystic ovarian syndrome, gut microbiota, probiotics, prebiotics, synbiotics

## Abstract

**Introduction:**

Accumulating evidence has highlighted the critical role of the gut microbiota and its potential action as a regulator of metabolic disorders including insulin resistance, obesity, and systemic inflammation in polycystic ovarian syndrome (PCOS). Microbiota-modulating interventions, such as probiotics, prebiotics, and synbiotics, could be effective in PCOS management.

**Methods:**

We conducted an overview of systematic reviews (SRs) and meta-analyses to summarize reviews regarding the effectiveness of probiotics/prebiotics/synbiotics on the management of PCOS through a systematic literature search in PubMed, Web of Science, and Scopus databases until September 2021.

**Results:**

Eight SRs and meta-analyses were included in this study. Our overview confirmed that probiotic supplementation had a potentially beneficial effect on some PCOS-related parameters including body mass index (BMI), fasting plasma glucose (FPG), and lipid profiles. Evidence shows that synbiotics in comparison with probiotics were less effective on these parameters. The methodological quality of SRs was assessed using the AMSTAR-2 assessment tool and was determined as high for four SRs, low for two SRs, and critically low for one SR. Due to limited evidence and high heterogeneity of the studies, it remains difficult to identify optimal probiotics strains, prebiotics types, length of duration, and doses.

**Discussion:**

Future clinical trials with higher quality are recommended to clarify the efficacy of probiotics/prebiotics/synbiotics on the management of PCOS and provide more accurate evidence.

## 1. Introduction

Polycystic ovarian syndrome (PCOS) is the most common endocrine disease in women of reproductive age, with an estimated prevalence of 6–26% worldwide. Increased awareness and screening have led to increasing diagnoses of this disease in adolescents. Although insulin resistance, impaired gonadotropin signaling, and altered ovarian reactivity have all been suggested for pathogenesis, the primary underlying cause of PCOS remains unknown (1). PCOS influences women's reproduction and increases the risk of long-term complications such as endometrial cancer, obesity, metabolic disease, dyslipidemia, and cardiovascular diseases (2). Therefore, effective treatment and management of PCOS have great importance in clinical and public health.

Gut microbiota and its beneficial role in the host's immunological, nutritional, and metabolic functions have been studied increasingly in the last decades (3–6). Since the gut microbiome has effects on different metabolic complications which are associated with PCOS including insulin resistance, obesity, and systemic inflammation, it might be involved in the pathogenesis of PCOS. Recent studies revealed that the gut microbiota of women with PCOS differs from that of healthy women. They reported a decrease in the overall bacterial species richness (α diversity) of the gut microbial community and changes in several bacterial taxa, in women with PCOS (7, 8). The main ways through which intestinal microbiota participates in the pathogenesis of PCOS are as follows: obesity leads to an imbalance in the intestinal flora, thus eradicating the connection between intestinal epithelial cells and expanding permeability of the gut mucosal which can cause leakage of lipopolysaccharide into the systemic circulation. These lead to the activation of the immune system and might influence the functioning of insulin receptors and cause insulin resistance. Hyperinsulinemia can increase the synthesis of testosterone, thus interfering the follicular development (9). Pharmacological treatments and lifestyle interventions are the classical options for the treatment of PCOS. However, these classical treatments are not effective in preventing the intergenerational transmission of PCOS and its associated metabolic dysfunction. Lifestyle interventions are proven effective in reducing the health risks of the offspring of patients with preconception PCOS in observational studies and preclinical animal models. Recent studies reported the association between PCOS pathogenesis and the gut microbiota. It was shown that the most common bacterial changes in PCOS patients consisted of *Bacteroides, Bacteroidaceae, Lactobacillus, Prevotella, Coprococcus, Parabacteroides, Escherichia/Shigella, and Faecalibacterium prausnitzii* (7, 10). Therefore, microbiota-modulating interventions, such as probiotics, prebiotics, and synbiotics, could be effective in PCOS management (11, 12). Probiotics, live microorganisms which have potential benefits when administered in adequate amounts, are proposed to improve the health of gut microflora by antagonizing the growth of pathogenic microorganisms, reducing gut leakiness and inflammation (13). Prebiotics are dietary non-digestible carbohydrates that may stimulate the growth and activity of beneficial microorganisms in the gut (14). Synbiotics are a mixture of both probiotics and prebiotics to support the survival of beneficial bacteria in the gut (15). These compounds have been suggested for application as a positive role in the host metabolism and can reduce pro-inflammatory markers and ameliorate blood lipid profiles and insulin resistance by the proliferation of the health-promoting bacteria such as *Bifidobacteria* and *Lactobacilli* and increasing the production of short-chain fatty acids (SCFAs) (16). These compounds can exert beneficial effects on body weight and metabolic profiles in PCOS through the gut–brain axis by activating satiety pathways, affecting the host's appetite (17), and modulation of the gastrointestinal immune system (18). There are many observational and interventional studies that investigated the effect of probiotics/prebiotics/synbiotics on PCOS management, and most of them support the use of these products in the prevention and treatment of PCOS, but sometimes there are some contradictions in their results. Moreover, many published systematic reviews or meta-analyses have focused on some specific metabolic outcomes. An overview of the published systematic reviews is a novel tool used to focus on specific issues related to policies and practices. The purpose is to synthesize the evidence from multiple systematic reviews into one available document, which can be used to guide healthcare professionals and decision-makers (19). We conducted this study to summarize and critically evaluate the evidence of systematic reviews regarding the effect of probiotics/prebiotics/synbiotics on the management of PCOS.

## 2. Materials and methods

### 2.1. Search strategy

A systematic literature search was conducted on PubMed, Web of Science, and Scopus databases. All related articles published up to 28 February 2023 were considered for inclusion. Search queries were as follows: (“Polycystic Ovary Syndrome” OR “Sclerocystic Ovarian Degeneration” OR “Polycystic ovary disease” OR “Sclerocystic Ovary Syndrome” OR “Sclerocystic Ovaries” OR “Polycystic Ovaries” OR “Stein Leventhal Syndrome” OR “Stein-Leventhal Syndrome”) AND (Prebiotic OR Prebiotics OR Probiotics OR Probiotic OR Synbiotics OR Synbiotic OR Parabiotic OR Parabiotics OR Postbiotics OR Postbiotic OR Lactobacillus OR Bifidobacterium OR Saccharomyces OR Inulin OR Dextrin OR FOS OR Fructooligosaccharide OR Galactooligosaccharide OR Lactulose OR Ligofructose OR Isomalt OR Microbiota OR Microbiome).

### 2.2. Eligibility criteria

Duplicate articles, which are articles retrieved from different queries, were removed, and only articles that had more complete data have been considered. Two researchers independently screened titles, abstracts, and full-text articles. Disagreements between the two researchers were resolved by discussion until reaching a consensus. Moreover, other relevant references of included articles were also reviewed. Studies were excluded if the main text was not in the English language. Original articles including observational and interventional studies, clinical trials, narrative reviews, protocols, editorials, letters, and case reports were also excluded. Therefore, only systematic reviews (SRs) and meta-analyses that investigated the effects of probiotics/prebiotics/synbiotics on any aspects of PCOS management were included in the present study.

The full text of the articles was analyzed to retrieve the relevant information including first author, published year, number and type of included studies in the SRs, total sample size, participants' characteristics, type, dose and duration of interventions, main outcomes, and reported side effects.

### 2.3. Assessing the quality of SRs

The methodological quality of the SRs was assessed using an AMSTAR-2 assessment tool (20). This tool is used for evaluating the methodological quality of SRs consisting of 16 items, of which, 7 are critical items, including the following items:

- Protocol registered before the commencement of the review (item 2);
- Adequacy of the literature search (item 4);
- Justification for excluding individual studies (item 7);
- Risk of bias from individual studies being included in the review (item 9);
- Appropriateness of meta-analytical methods (item 11);
- Consideration of risk of bias when interpreting the results of the review (item 13); and
- Assessment of presence and likely impact of publication bias (item 15).

Each item can be evaluated as yes, partial yes, or no. According to the number of violations of key items, the quality of research is divided into four levels as follows: high, medium, low, or extremely low.

## 3. Results

**Figure 1:**
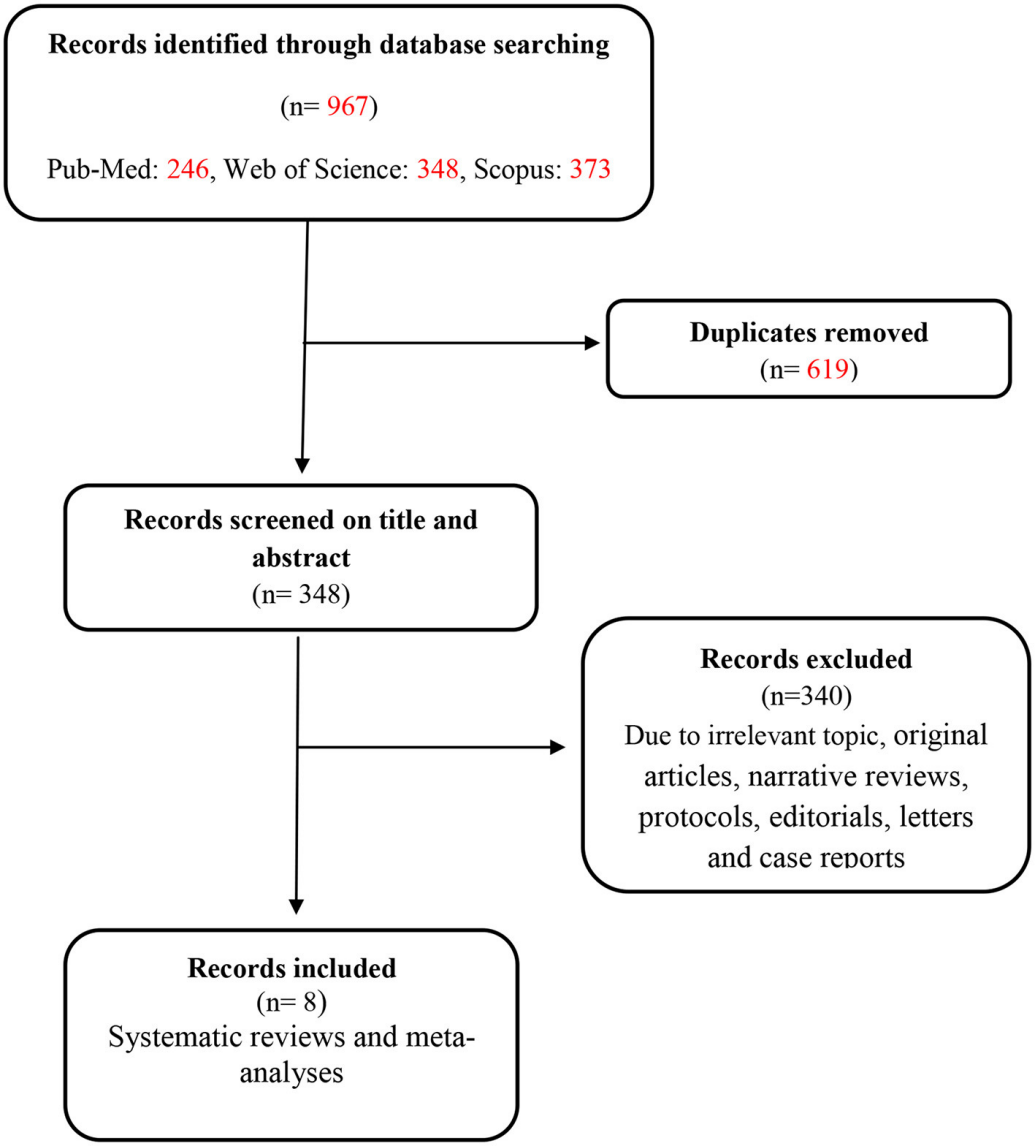
Flowchart of studies identified.

As shown in the flowchart in Figure 1, a total of 967 records were initially retrieved from three databases (PubMed: 246, Web of Science: 348, and Scopus: 373). After the removal of duplicates, 348 records were screened by title and abstract. In total, 340 studies were excluded during the screening of full text due to the irrelevance of the subject, being observational or interventional studies, narrative reviews, protocols, editorials, letters, case reports, and missing outcome data. Finally, eight articles were obtained for this study. Among these eight included documents, we did not have access to the full text of one article (21).

### 3.1. Study characteristics

The eight included SRs and meta-analyses were published between 2018 and 2023. All meta-analyses evaluated the probiotics and synbiotics effects on PCOS-related parameters, and two of them evaluated the prebiotics effects too. The number of randomized clinical trials (RCTs) as original studies in these SRs ranged from 6 to 17, with sample sizes ranging from 406 to 1,049. The duration of included RCTs in these SRs ranged from 8 to 12 weeks. SRs mainly involve 16 outcomes, including body mass index (BMI), fasting plasma glucose (FPG), fasting blood insulin (FBI), triglycerides (TG), total cholesterol (TC), low-density lipoprotein-cholesterol (LDL-C), very low-density lipoprotein-cholesterol (VLDL-C), high-density lipoprotein-cholesterol (HDL-C), quantitative insulin sensitivity check index (QUICKI), C-reactive protein (CRP), nitric oxide (NO), total antioxidant capacity (TAC), total glutathione (GSH), malondialdehyde (MDA), sex hormone-binding globulin (SHBG), and free androgen index (FAI). The basic characteristics of the included SRs are shown in Table 1.

**Table 1:** Baseline characteristics of included meta-analyses investigating the effects of probiotics and synbiotics on PCOS.

**References**	**Included studies in meta-analysis (*n*)**	**Type of included studies in metaanalysis**	**Total sample size (*n*)**	**Participants' characteristics**	**Type and dose of intervention**	**Duration of interventions (weeks)**	**Main outcomes significant**	**Risk of bias assessment**	**Reported side effects**	**AMSTAR score**
Hadi et al. (22)	8	RCTs (blinding, paralel)	540	Women with PCOS (25–30 years)	Live bacteria as probiotics or synbiotics (not in combination with other drugs or supplements) Lactobacillus, Bifidobacterium (2 × 10^7^-2 × 10^10^) Inulin (0.8–20 gr) Placebo(starch, MDX)	8–12 weeks	↓FBS ↓Insulin ↓HOMA I-R ↓CRP ↓Total testosterone	Yes	No	Critically low
Miao et al. (23)	7 (6 in iran and 1 in china)	RCTs	486	Women with PCOS	Probiotics and synbiotics Lactobacillus, Bacillus, Bifidobacterium, metformin as a co-intervention	8–12 weeks	↓HOMA I-R ↓Insulin	Yes	No	High
Li et al. (24)	17 (15 in iran and 2 in china)	RCTs	1,049	Women with PCOS	Probiotics, prebiotics, and synbiotics (intake separately or in combination with other drugs, compared with placebo) 1 × 10^7^ to 10^10^	8–12 weeks	↓FPG fasting insulin ↓HOMA I-R ↓TG ↓Total cholesterol ↓LDL ↓VLDL-C ↑QUICKI	Yes	No	High
Tabrizi et al. (25)	11	RCTs	730	Women with PCOS (24–30 years)	Probiotic and/or symbiotic capsule contained: Lactobacillus, Bifidobacterium 2 × 10^8^ to 2 × 10^10^	8–12 weeks	↓Weight ↓BMI ↓FPG ↓ Insulin ↓HOMA I-R ↓TG ↓VLDL-C ↓CRP ↓MDA ↓Hirsutism ↓Total testosterone levels ↑QUICKI ↑NO ↑TAC ↑GSH ↑ SHBG	Yes	No	High
Shamasbi et al. (26)	13	RCTs	855	Women with PCOS (15–49 years)	Probiotics, prebiotics, and synbiotics Lactobacillus, Bifidobacterium (powder or capsule different doses) comparison group included the placebo or maltodextrin group	8–12 weeks	↑ SHBG ↑NO ↓FAI ↓MDA	Yes	No	Low
Heshmati et al. (27)	7	RCTs	471 (236 women with PCOS and 235 controls)	Women with PCOS (25–30 years)	Probiotics (or synbiotics) and the placebo groups 2 × 10^9^/complex	8–12 weeks	↑QUICKI ↓TG level ↓Fasting insulin ↓HDL	Yes	No	Low
Cozzolino et al. (28)	9	RCTs (Eight studies were double-blinded, whilst one study was triple-blinde)	587	Women with PCOS	Probiotic/symbiotic (Control group women with PCOS without therapy with probiotics or synbiotics or placebo) Lactobacillus, Bifidobacterium 2 × 10^8^ to 3 × 10^10^	8 Cozzolino 12 weeks	↓FPG ↓FBI ↓HOMA I-R ↓BMI ↓Serum TG ↓Serum testosterone ↑hs-CRP ↑NO ↑TAC ↑GSH ↑MDA	Yes	No	High
Liao et al. (21)	6	RCTs	406	Women with PCOS aged 25–28.5 years	Probiotic supplementation	8–12 weeks	↑QUICKI ↓FBI ↓TG ↓VLDL-C	Yes	No	NA

FBS, fasting blood sugar; CRP, C reactive protein; hsCRP, high sensitive C reactive protein; BMI, Body mass index; FPG, fasting plasma glucose; SHBG, sex hormone binding globulin; NO, nitric oxide; FBI, fasting blood insulin; TG, triglycerides; QUICKI, quantitative insulin sensitivity check index; TAC, total antioxidant capacity; GSH, total glutathione; MDA, malondialdehyde; FAI, Free Androgen Index; VLDL-C, very low density lipoprotein-cholesterol; HDL-C, high density lipoprotein-cholesterol; HOMA I-R, homeostatic model assessment-insulin resistance.

### 3.2. AMSTAR-2 evaluation results

The methodological quality was evaluated as high for four SRs (23–25, 28), low for two SRs (26, 27), and critically low for one SR (22). Among the seven key items of the AMSTAR-2 tool, six items (2, 4, 5, 7, 9, 11, and 13) were relatively complete and were reported by ≥85% of the SRs, while item 15 (assessment of publication bias) was not reported by 3 SRs (22, 26, 27). Details of evaluation results are presented in Table 2.

**Table 2:** Quality scores of included meta-analyses investigating the effects of probiotics and synbiotics supplementation on PCOS.

**References**	**AMSTAR ITEMS** ^*^																
	**1**	**2**	**3**	**4**	**5**	**6**	**7**	**8**	**9**	**10**	**11**	**12**	**13**	**14**	**15**	**16**	**Score**
Hadi et al. (22)	Yes	Partial Yes	Yes	Partial Yes	Yes	No	No	Partial Yes	Partial Yes	Yes	Yes	Yes	Yes	No	No	Yes	Critically low
Miao et al. (23)	Yes	Yes	Yes	Partial Yes	Yes	Yes	Yes	Yes	Yes	Yes	Yes	Yes	Yes	Yes	Yes	Yes	High
Li et al. (24)	Yes	Yes	Yes	Partial Yes	Yes	Yes	Yes	Yes	Yes	Yes	Yes	Yes	Yes	Yes	Yes	Yes	High
Tabrizi et al. (25)	Yes	Partial Yes	Yes	Partial Yes	Yes	No	Yes	Yes	Yes	Yes	Yes	Yes	Yes	Yes	Yes	Yes	High
Shamasbi et al. (26)	Yes	Partial Yes	Yes	Partial Yes	Yes	Yes	Yes	Yes	Yes	Yes	Yes	Yes	Yes	No	No	Yes	Low
Heshmati et al. (27)	Yes	Partial Yes	Yes	Partial Yes	Yes	Yes	Yes	Yes	Yes	No	Yes	Yes	Yes	Yes	No	Yes	Low
Cozzolino et al. (28)	Yes	Yes	Yes	Partial Yes	Yes	Yes	Yes	Yes	Yes	Yes	Yes	Yes	Yes	No	Yes	Yes	High

* Since we have no access to the full text of Liao et al. (2018), we cannot do quality assessment for this study.

### 3.3. Effects of probiotics, prebiotics, and synbiotics on PCOS

The main findings of included meta-analyses investigating the overall and subgroup effects of probiotic and synbiotic supplementation on PCOS are presented in Table 3.

**Table 3:** Main findings of included meta-analyses investigating the overall and subgroup effects of probiotics and synbiotics supplementation on PCOS.

**Health indicator group**	**Health indicator**	**Number of meta-analyses**	**References**	**Number of original articles (number of participants)**	**Mean difference [95% CI]**	**Significance**	**Heterogenicity**
**Probiotics/synbiotics**							
Anthropometrics	BMI	5	Hadi et al. (22)	7 (386)	−0.23 [−0.55, 0.08]	No	*I*^2^ = 92.6
			Li et al. (24)	13 (791)	−0.10 [−0.30, 0.09]	No	*I*^2^ = 47.3
			Tabrizi et al. (25)	9 (506)	−0.29 [−0.54, – 0.03]	Yes	*I*^2^ = 50.7
			Cozzolino et al. (28)	7 (416)	−0.25 [−0.48, −0.03]	Yes	*I*^2^ = 97
			Miao et al. (23)	4 (262)	−0.74 [−1.58, 0.11]	No	*I*^2^ = 0
	BW	5	Li et al. (24)	12 (731)	−0.11 [−0.34, 0.13]	No	*I*^2^ = 61.5
			Hadi et al. (22)	7 (386)	−0.67 [−1.43, 0.10]	No	*I*^2^ = 90.8
			Tabrizi et al. (25)	9 (506)	– 0.30 (−0.53, −0.07]	Yes	*I*^2^ = 42.5
			Cozzolino et al. (28)	7 (416)	−0.75 [−1.45, −0.05]	Yes	*I*^2^ = 97
			Li et al. (24)	5 (316)	0.37 [−0.78, 1.53]	No	*I*^2^ = 95.5
	HC	1	Li et al. (24)	4 (256)	−0.25 [−0.78, 0.27]	No	*I*^2^ = 76.9
Lipids	HDL-C	5	Li et al. (24)	7 (428)	0.53 [−0.33, 1.39]	N0	*I*^2^ = 94.3
			Tabrizi et al. (25)	3 (180)	0.04 [– 0.25, 0.33]	No	*I*^2^ = 0
			Heshmati et al. (27)	3 (119)	1.55 [0.28, 2.81]	Yes	*I*^2^ = 0
	LDL-C		Tabrizi et al. (25)	3 (180)	– 0.12 [– 0.66, 0.42]	No	*I*^2^ =70.2
	VLDL-C	2	Li et al. (24)	7 (428)	−0.84 [−1.64, −0.03]	Yes	*I*^2^ = 93.4
			Li et al. (24)	4 (235)	−0.44 [−0.70, −0.18]	Yes	*I*^2^ = 0
			Tabrizi et al. (25)	3 (180)	– 0.69 [−0.99, – 0.39]	Yes	*I*^2^ = 0
	TC	2	Heshmati et al. (27)	3 (208)	0.99 [−5.31, 7.29]	No	*I*^2^ = 0
			Tabrizi et al. (25)	3 (180)	– 0.26 [– 0.67, 0.15]	No	*I*^2^ = 48.4
	TG	4	Heshmati et al. (27)	3 (119)	−17.51 [−29.65, −5.36]	Yes	*I*^2^ = 32
			Cozzolino et al. (28)	2 (120)	−23.35 [−35.23, −11.47]	Yes	*I*^2^ = 0
			Li et al. (24)	7 (428)	−0.85 [−1.59, −0.11]	Yes	*I*^2^ = 92.2
			Tabrizi et al. (25)	3 (180)	– 0.69 [– 0.99, – 0.39]	Yes	*I*^2^ = 0
Glucose homeostasis	FBS	2	Hadi et al. (22)	6 (360)	−2.52 [−4.10, −0.95]	Yes	*I*^2^ = 0
			Miao et al. (23)	4 (265)	−1.94 [−5.53, 1.65]	No	*I*^2^ = 91
	FPG	4	Heshmati et al. (27)	4 (291)	−3.38 [−7.08, 0.31]	Yes	*I*^2^ = 81
			Cozzolino et al. (28)	5 (337)	−3.45 [−6.03, −0.88]	Yes	*I*^2^ = 84
			Li et al. (24)	8 (496)	−1.35 [−2.22,−0.49]	Yes	*I*^2^ = 94.6
			Tabrizi et al. (25)	7 (430)	– 0.26 [−0.45, – 0.07]	Yes	*I*^2^ = 0
	HOMA-IR	6	Hadi et al. (22)	6 (360)	−0.69 [−0.98, −0.40]	Yes	*I*^2^ = 40.2
			Heshmati et al. (27)	4 (291)	−0.48 [−0.97, −0.02]	No	*I*^2^ = 74
			Cozzolino et al. (28)	5 (337)	−2.31 [−3.84, −0.77]	Yes	*I*^2^ = 94
			Miao et al. (23)	5 (325)	−0.37 [−0.69, −0.05]	Yes	*I*^2^ = 58
			Li et al. (24)	7 (434)	−0.73 [−1.15, −0.31]	Yes	*I*^2^ = 78.1
			Tabrizi et al. (25)	7 (430)	– 0.53 [– 0.79, – 0.26]	Yes	*I*^2^ = 44.5
	FBI	4	Hadi et al. (22)	6 (360)	−2.27 [−3.40, −1.14]	Yes	*I*^2^ = 42.9
			Heshmati et al. (27)	4 (291)	−2.14 [−4.24, −0.04]	Yes	*I*^2^ = 73
			Li et al. (24)	7 (434)	−0.68 [−1.08, −0.27]	Yes	*I*^2^ = 76.7
			Tabrizi et al. (25)	7 (430)	– 0.52 [– 0.81, – 0.24]	Yes	*I*^2^ = 52.2
	QUICKI	4	Heshmati et al. (27)	4 (291)	0.41 [0.01, 0.82]	Yes	*I*^2^ = 66
			Cozzolino et al. (28)	5 (337)	−0.62 [−1.07, −0.17]	Yes	*I*^2^ = 89
			Li et al. (24)	6 (379)	2.00 [−0.79, 3.22]	Yes	*I*^2^ = 96.1
			Tabrizi et al. (25)	7 (430)	0.41 [0.11, 0.70]	Yes	*I*^2^ = 55.5
Inflammation and antioxidant	GSH	3	Shamasbi et al. (26)	3 (180)	0.53 [−0.00, 1.06]	Yes	*I*^2^ = 68
			Cozzolino et al. (28)	3 (180)	22.42 [2.08, 42.75]	Yes	*I*^2^ = 25
			Tabrizi et al. (25)	4 (240)	0.26 [0.01, 0.52]	Yes	*I*^2^ = 0
	MDA	2	Shamasbi et al. (26)	3 (180)	−0.76 [−1/46,−0.05]	Yes	*I*^2^ = 81
			Tabrizi et al. (25)	4 (240)	– 0.90 [−1.16, – 0.63]	Yes	*I*^2^ = 0
	NO	3	Shamasbi et al. (26)	3 (180)	0.38 [0.09, 0.68]	Yes	*I*^2^ = 0
			Cozzolino et al. (28)	3 (180)	2.72 [0.08, 0.59]	Yes	*I*^2^ = 26
			Tabrizi et al. (25)	4 (240)	0.33 [– 2.14, – 0.37]	Yes	*I*^2^ = 0
	CRP	4	Heshmati et al. (27)	2 (153)	0.92 [−0.57, 2.40]	No	*I*^2^ = 73
			Li et al. (24)	9 (558)	−0.63 [−1.37, 0.10]	No	*I*^2^ = 93.9
			Tabrizi et al. (25)	7 (464)	– 1.26 [– 2.14, – 0.37]	Yes	*I*^2^ = 94.6
			Hadi et al. (22)	5 (334)	−1.69 [−3.00, −0.38]	Yes	*I*^2^ = 96.5
	hsCRP	3	Shamasbi et al. (26)	8 (567)	−0.59 [−1.60, 0.42]	No	*I*^2^ = 96
			Heshmati et al. (27)	3 (180)	−0.00 [−1.46, 1.46]	No	*I*^2^ = 95
			Cozzolino et al. (28)	4 (240)	−1.69 [−2.38, −1.01]	Yes	*I*^2^ = 49
	TAC	3	Shamasbi et al. (26)	3 (180)	0.30 [−0.58, 1.17]	No	*I*^2^ = 88
			Cozzolino et al. (28)	3 (180)	70.55 [38.84, 102.25]	Yes	I^2^=25
			Tabrizi et al. (25)	4 (240)	0.64 [0.38, 0.90]	Yes	*I*^2^ = 0
Hormones	SHBG	2	Shamasbi et al. (26)	3 (180)	0.56 (0.26, 0.86]	Yes	*I*^2^ = 0
			Tabrizi et al. (25)	4 (240)	0.46 [0.08, 0.85]	Yes	*I*^2^ =55.7
	DHEA-S	2	Shamasbi et al. (26)	3 (182)	−0.22 [−0.51, 0.07]	No	*I*^2^ = 0
			Tabrizi et al. (25)	2 (120)	0.06 [– 0.77, 0.89]	No	*I*^2^ =80.8
	Total testosteron	4	Hadi et al. (22)	4 (206)	−0.12 [−0.17, −0.08]	Yes	*I*^2^ = 43.4
			Shamasbi et al. (26)	3 (180)	−0.50 [−1.25, 0.25]	No	*I*^2^ = 84
			Cozzolino et al. (28)	4 (226)	−0.23 [−0.36, −0.11]	Yes	*I*^2^ = 52
			Tabrizi et al. (25)	6 (326)	– 0.58 [– 0.82, – 0.34]	Yes	*I*^2^ = 10.4
	FAI	1	Shamasbi et al. (26)	2 (120)	−0.58 [−0.95, 00.21]	Yes	*I*^2^ = 68
Clinical symptoms	Hirsutism	1	Shamasbi et al. (26)	4 (242)	−0.12 [−0.38, 0.13]	No	*I*^2^ = 50
**Synbiotics**							
Anthropometrics	BMI	4	Hadi et al. (22)	4 (206)	−0.03 [−0.25, 0.19]	No	*I*^2^ = 43.2
			Li et al. (24)	4 (254)	−0.13 [−0.53, 0.26]	No	*I*^2^ = 58.90
			Tabrizi et al. (25)	4	– 0.03 [– 0.30, 0.25]	*I*^2^ = 0.0
			Cozzolino et al. (28)	3 (166)	−0.19 [−0.74, 0.36]	No	*I*^2^ = 98^*^
	BW	3	Hadi et al. (22)	4 (206)	−0.08 [−0.58, 0.39]	No	*I*^2^ = 17.9
			Cozzolino et al. (28)	3 (166)	−0.66 [−2.31, 0.98]	No	*I*^2^ = 98^*^
			Li et al. (24)	4 (254)	0.12 [−0.49, 0.25]	No	*I*^2^ = 53.50
	WC	2	Miao et al. (23)	3 (207)	−1.88 [−4.88, 1.12]	No	*I*^2^ = 63^*^
			Li et al. (24)	2 (134)	−0.48 [−1.52, 0.56]	No	*I*^2^ = 87.20
	HC	1	Li et al. (24)	2 (134)	−0.21 [−0.92, 0.50]	No	*I*^2^ = 74.20
Lipids	HDL-C	2	Li et al. (24)	3 (191)	0.09 [−0.48, 0.65]	No	*I*^2^ = 72.70
			Heshmati et al. (27)	2 (159)	1.64 [0.33, 2.94]	Yes	*I*^2^ = 0^*^
	LDL–C	3	Heshmati et al. (27)	2 (159)	−5.59 [−9.58, −1.61]	Yes	*I*^2^ = 0
			Tabrizi et al. (25)	1	– 0.07 [– 0.57, 0.44]	NA
			Li et al. (24)	3 (191)	−0.22 [−0.51, 0.06]	No	*I*^2^ = 0
	VLDL–c	1	Li et al. (24)	1 (60)	−0.32 [−0.83, 0.19]	No	NA
	TC	2	Heshmati et al. (27)	2 (148)	−0.22 [−9.41, 8.97]	No	*I*^2^ = 32
			Li et al. (24)	3 (191)	−0.28 [−0.56, 0.01]	No	*I*^2^ = 0
	TG	2	Heshmati et al. (27)	2 (159)	−14.54 [−30.54, 1.45]	Yes	*I*^2^ = 53
			Li et al. (24)	3 (191)	−0.14 [−0.47, 0.20]	No	*I*^2^ = 25.20
Glucose homeostasis	FBS	1	Hadi et al. (22)	4 (345)	−2.05 [−3.79, −0.31]	Yes	*I*^2^ = 0
	FPG	3	Heshmati et al. (27)	2 (159)	−0.77 [−4.53, 2.99]	No	*I*^2^ = 82
			Cozzolino et al. (28)	3 (205)	−1.50 [−2.03, −0.98]	Yes	*I*^2^ = 0^*^
			Li et al. (24)	3 (194)	−0.36 [−0.87, 0.15]	No	*I*^2^ = 67.70
	HOMA-IR	4	Hadi et al. (22)	4 (245)	−0.82 [−1.10, −0.53]	Yes	*I*^2^ = 14.1
			Li et al. (24)	3 (194)	−0.74 [−1.59, 0.11]	No	*I*^2^ = 87.20
			Heshmati et al. (27)	2 (159)	−0.50 [−1.72, 0.71]	No	*I*^2^ = 77
			Cozzolino et al. (28)	3 (205)	−2.75 [−4.56, −0.95]	Yes	*I*^2^ = 55^*^
	FBI	5	Hadi et al. (22)	4 (245)	−2.40 [−3.81, −0.99]	Yes	*I*^2^ = 49.7
			Li et al. (24)	3 (194)	−0.67 [−1.54, 0.20]	No	*I*^2^ = 87.90
			Heshmati et al. (27)	2 (159)	−2.38 [−6.96, 2.20]	No	*I*^2^ = 77
			Miao et al. (23)	4 (270)	−0.66 [−1.19, −0.12]	Yes	*I*^2^ = 78^*^
			Tabrizi et al. (25)	4	– 0.50 [−0.93, −0.06]	*I*^2^ = 63.5
	QUICKI	4	Heshmati et al. (27)	2 (159)	0.43 [−0.42, 1.28]	No	*I*^2^ = 85
			Li et al. (24)	3 (194)	0.92 [−0.12, 1.96]	No	*I*^2^ = 91
			Cozzolino et al. (28)	3 (205)	−0.80 [−1.20, −0.39]	Yes	*I*^2^ = 46^*^
			Tabrizi et al. (25)	4	0.44[– 0.05, 0.92]	*I*^2^ = 70.2
Inflammation and antioxidant	GSH	1	Shamasbi et al. (26)	1 (60)	0.02 [−0.48, 0.53]	No	Na
	MDA	1	Shamasbi et al. (26)	1 (60)	−0.66 [−1.18, −0.14]	Yes	NA
	NO	1	Shamasbi et al. (26)	1 (60)	0.49 [−0.02, 1.00]	No	Na
	CRP	3	Hadi et al. (22)	2 (159)	−0.51 [−0.83, −0.20]	Yes	*I*^2^ = 0
			Li et al. (24)	3 (191)	−0.50 [−1.16, 0.15]	No	*I*^2^ = 79.10
			Tabrizi et al. (25)	2	0.52 [– 0.83, – 0.20]	*I*^2^ = 0
	hsCRP	1	Shamasbi et al. (26)	2 (159)	−0.47 [−1.97, 1.03]	No	*I*^2^ = 95
	TAC	1	Shamasbi et al. (26)	1 (60)	−0.52 [−1.03, −0.00]	Yes	Na
Hormones	SHBG	1	Shamasbi et al. (26)	1 (60)	0.49 [−0.02, 1.01]	No	Na
	DHEA-S	1	Shamasbi et al. (26)	1 (60)	−0.31 [−0.82, 0.20]	No	Na
	Total testosteron	2	Shamasbi et al. (26)	1 (60)	0.10 [−0.40, 0.61]	No	Na
			Cozzolino et al. (28)	2 (106)	−0.15 [−0.16, −0.14]	Yes	I^2^ = 0
	FAI	1	Shamasbi et al. (26)	1 (60)	−0.26 [−0.77, 0.25]	No	Na
Clinical symptoms	Hirsutism	1	Shamasbi et al. (26)	1 (60)	−0.23 [−0/74, 0.28]	No	NA (not applicable)
**Probiotics**							
Anthropometrics	BMI	5	Hadi et al. (22)	3 (180)	−0.36 [−0.74, 0.02]	No	*I*^2^ = 91.3
			Li et al. (24)	8 (475)	−0.03 [−0.24, 0.19]	No	*I*^2^ = 31.80
			Tabrizi et al. (25)	5	−0.29 [– 0.79, – 0.14]	Yes	*I*^2^ = 48.6
			Cozzolino et al. (28)	4 (250)	−0.31 [−0.65, 0.03]	Yes	*I*^2^ = 96
	BW	3	Li et al. (24)	7 (415)	−0.02 [−0.36, 0.31]	No	*I*^2^ = 66.20
			Hadi et al. (22)	3 (180)	−1.3 [−1.93, −0.13]	Yes	*I*^2^ = 90.6
			Cozzolino et al. (28)	4 (250)	−0.80 [−1.76, 0.15]	No	*I*^2^ = 97
	WC	1	Li et al. (24)	2 (120)	1.87[−0.91, 4.65]	No	*I*^2^ = 97.20
	HC	1	Li et al. (24)	1 (60)	0.25 [−0.26, 0.75]	No	NA
Lipids	HDL-C	3	Li et al. (24)	3 (175)	−0.17 [−0.98, 0.63]	No	*I*^2^ = 85.90
			Li et al. (24)	3 (175)	−0.17 [−0.98, 0.63]	NO	*I*^2^ = 85.90
			Heshmati et al. (27)	1 (60)	−0.10 [−5.60, 5.40]	No	Na
	LDL-C	3	Heshmati et al. (27)	1 (60)	8.30 [−4.50, 21.10]	No	Na
			Li et al. (24)	3 (175)	−0.13 [−0.42, 0.17]	NO	*I*^2^ = 0
			Tabrizi et al. (25)	2	– 0.15 [−1.09, 0.79]	Yes	*I*^2^ = 85
	VLDL-c	1	Li et al. (24)	3 (175)	−0.48 [−0.78, −0.18]	YES	*I*^2^ = 0
	TC	2	Heshmati et al. (27)	1 (60)	2.80 [−11.36, 16.96]	No	Na
			Li et al. (24)	3 (175)	−0.26 [−0.85, 0.32]	Yes	*I*^2^ = 73.50
	TG	2	Heshmati et al. (27)	1 (60)	−26.90 [−49.55, −4.25]	Yes	Na
			Li et al. (24)	3 (175)	−0.50 [−0.80, −0.20]	Yes	*I*^2^ = 0
Glucose homeostasis	FBS	1	Hadi et al. (22)	2 (120)	−4.70 [−8.43, −0.96]	Yes	*I*^2^ = 0
	FPG	3	Heshmati et al. (27)	2 (132)	−6.23 [−8.07, −4.40]	Yes	*I*^2^ = 0
			Cozzolino et al. (28)	2 (132)	−6.23 [−8.07, −4.40]	Yes	*I*^2^ = 0
			Li et al. (24)	4 (240)	−0.96 [−1.86, −0.07]	Yes	*I*^2^ = 90.50
	HOMA-IR	4	Hadi et al. (22)	2 (115)	−0.47 [−1.05, 0.12]	No	*I*^2^= 52.9
			Li et al. (24)	4 (240)	−0.74 [1.25, 0.23]	Yes	*I*^2^ = 73.10
			Heshmati et al. (27)	2 (132)	−0.41 [−0.98, 0.15]	No	*I*^2^ = 70
			Cozzolino et al. (28)	2 (132)	−1.87 [−4.50, 0.76]	No	*I*^2^ = 74
	FBI	4	Hadi et al. (22)	2 (115)	−2.07 [−4.76, 0.63]	No	*I*^2^ = 59.8
			Li et al. (24)	4 (240)	−0.70 [−1.13, −0.26]	Yes	*I*^2^ = 63.60
			Tabrizi et al. (25)	3	– 0.57 [– 0.98, – 0.16]	Yes	*I*^2^ = 47.5
			Heshmati et al. (27)	2 (132)	−1.87 [−4.50, 0.76]	No	*I*^2^ = 74
	QUICKI	3	Heshmati et al. (27)	2 (132)	0.42 [−0.07, 0.91]	No	*I*^2^ = 50
			Li et al. (24)	3 (185)	3.65 [0.71, 6.58]	Yes	*I*^2^ = 98.10
			Tabrizi et al. (25)	3	0.38 [0.01, 0.77]	Yes	*I*^2^ = 41.3
			Cozzolino et al. (28)	2 (132)	−0.41 [−0.98, 0.15]	No	*I*^2^ = 70
Inflammation and antioxidant	GSH	1	Shamasbi et al. (26)	2 (120)	−0.81 [−2.04, 0.42]	No	NA
	MDA	1	Shamasbi et al. (26)	2 (120)	0.78 [0.41, 1.15]	Yes	*I*^2^ = 0
	NO	1	Shamasbi et al. (26)	2 (120)	0.33 [−0.03, 0.69]	Yes	*I*^2^ = 0
	CRP	32	Hadi et al. (22)	3 (175)	−2.80 [−5.75, 0.15]	No	*I*^2^ = 98.2
			Tabrizi et al. (25)	5	– 1.73[– 3.13, – 0.33]	Yes	I^2^ = 96.4
			Li et al. (24)	5 (305)	−0.12 [−1.01, 0.77]	No	I^2^=92.90
	hsCRP	1	Shamasbi et al. (26)	5 (346)	0.09 [−1.13, 1.30]	No	*I*^2^ = 95
	TAC	1	Shamasbi et al. (26)	2 (120)	0.70 [0.08, 1.32]	Yes	*I*^2^ = 64
Hormones	SHBG	1	Shamasbi et al. (26)	2 (120)	0.59 [0.23, 0.96]	Yes	*I*^2^ = 0
	DHEA-S	1	Shamasbi et al. (26)	1 (60)	0.00 [−0.51, 0.51]	No	Na
	Total testosteron	2	Shamasbi et al. (26)	2 (120)	−0.80 [−1.63, 0.03]	No	*I*^2^ = 79
			Cozzolino et al. (28)	2 (120)	−0.32 [−0.46, −0.18]	Yes	*I*^2^ = 0
	FAI	1	Shamasbi et al. (26)	1 (60)	−0.93 [−1.46, −0.39]	Yes	Na
Clinical symptoms	hirsutism	1	Shamasbi et al. (26)	2 (120)	0.15 [−0/21, 0.51]	No	*I*^2^ = 0

BMI, Body mass index; CRP, C reactive protein; FAI, Free Androgen Index; FBS: fasting blood sugar; FPG, fasting plasma glucose; FBI, fasting blood insulin; GSH, total glutathione; HC, hip circumference; HDL-C, high density lipoprotein-cholesterol; HOMA-IR, homeostatic model assessment-insulin resistance; hsCRP, high sensitive C reactive protein; MDA, malondialdehyde; NO, nitric oxide; QUICKI, quantitative insulin sensitivity check index; SHBG, sex hormone binding globulin; TG, triglycerides; TAC, total antioxidant capacity; VLDL-C, very low density lipoprotein-cholesterol.

#### 3.3.1. Effects of probiotics, prebiotics, and synbiotics on anthropometric indices

The effectiveness of probiotics on BMI was evaluated in four meta-analyses (22, 24, 25, 28), **two** of which showed that probiotic supplementation significantly decreased BMI (25, 28). Standardized mean difference (SMD) was reported as −0.031 kg/m^2^ by Cozzalino et al. and −0.29 kg/m^2^ by Tabrizi et al. (18, 19). Other anthropometric parameters including waist circumference (WC) and hip circumference (HC) were evaluated in one meta-analysis conducted by Li Y. et al., and no significant alteration was reported by probiotic supplementation. However, in this study, the administration of prebiotics led to significantly decreased BMI (SMD:-0.66 kg/m^2^), WC (SMD:-0.76 cm), and HC (SMD:-0.85 cm) (21, 24). The effectiveness of synbiotics on anthropometric parameters was assessed in four meta-analyses, and no significant alteration was reported (22, 24, 25, 28).

#### 3.3.2. Effects of probiotics, prebiotics, and synbiotics on lipid profiles

The effectiveness of probiotics on lipid profiles was evaluated in four meta-analyses (21, 23, 25, 27). All of them showed that probiotic supplementation significantly decreased TG (range of reported SMD: −0.50 to −0.69 mg/dL) but had no effect on HDL-C and LDL-C. Li, Y. et al. and Liao reported a significant decrease in VLDL-C by probiotic supplementation (21, 24). The effectiveness of synbiotics on lipid profiles was assessed in two meta-analyses, one of which showed that the administration of synbiotics led to both significantly decreased TG (mean difference (MD): −14.54 mg/dL) and LDL-C (MD: −5.59 mg/dL) and significantly increased HDL-C (MD: 1.64 mg/dL) (27), but Li Y. et al. reported no significant alteration (24).

#### 3.3.3. Effects of probiotics, prebiotics, and synbiotics on glucose homeostasis

The effectiveness of probiotics on glucose homeostasis was evaluated in six meta-analyses, and all of them showed that probiotic supplementation significantly decreased FPG (range of reported MD: −0.26 to −6.33 mg/dL) (21, 22, 24, 25, 27, 28). Two meta-analyses reported a significant positive effect on insulin resistance indices, such as FBI (range of reported MD: −0.57 to −0.70 mg/dL) and QUICKI (range of reported MD: 0.38 to 3.65) (24, 25). The effectiveness of prebiotics on FPG was evaluated in one meta-analysis which showed a significant reduction (MD:-6.98 mg/dL) (24). In addition, the effectiveness of synbiotics on glucose homeostasis was evaluated in six meta-analyses. Two of them reported a significant reduction in FPG (range of reported MD: −1.50 to −2.05 mg/dL) and HOMA-IR (range of reported MD: −0.82 to −2.75) (22, 28).

#### 3.3.4. Effects of probiotics, prebiotics, and synbiotics on inflammation and oxidative stress

The effectiveness of probiotics and synbiotics on some inflammation and antioxidant indices including GSH, MDA, NO, TAC, and CRP was assessed in five meta-analyses. Significant positive effects were observed on GSH (range of reported MD: 0.26–22.42) (25, 26, 28), MDA (range of reported MD: −0.76 to −0.90 μmol/L) (25, 26), NO (range of reported MD: 0.33 to 2.72 μM) (25, 26, 28), TAC (range of reported MD: 0.64–70.55) (25, 28), and CRP (range of reported MD: −1.26 to −1.69 mg/L) (22, 25, 28).

#### 3.3.5. Effects of probiotics, prebiotics, and synbiotics on sexual hormones and clinical symptoms

The effectiveness of probiotics, prebiotics, and synbiotics on sexual hormones and clinical symptoms was assessed in one meta-analysis conducted by Shamasbi et al. The probiotics and synbiotics supplementation had a significant positive effect on SHBG (SMD: 0.56 nmol/L) and FAI (SMD: −0.58) but had no effect on DHEA-S, total testosterone, and hirsutism (26). Cozzolino et al. reported a significant reduction in total testosterone by both probiotics and synbiotics administration (28).

## 4. Discussion

This is the first overview of systematic reviews to comprehensively evaluate the efficacy of probiotics, prebiotics, and synbiotics supplementation on PCOS. We identified eight SRs with meta-analysis in this field, covering 5,247 participants in RCTs. Based on the AMSTAR-2 assessment tool, the quality of most included SRs was high. Specifically, in key items, most included SRs showed positive results which decrease the risk of bias and make them more reliable. According to the existing evidence, our review confirmed that probiotics had a potentially beneficial effect on some PCOS-related parameters including BMI, FPG, and lipid profiles. Synbiotics in comparison with probiotics were less effective on anthropometric parameters, lipid profiles, and glucose hemostasis. There are insufficient studies regarding the effectiveness of prebiotics on PCOS-related parameters, but one meta-analysis with high quality has supported the beneficial effects of prebiotics on anthropometric parameters, FPG, and CRP (24). Concerning the effectiveness of probiotics/prebiotics/synbiotics on inflammatory indices, sexual hormones, and clinical symptoms, a few systematic reviews have been conducted; however, the results were relatively favorable. Additionally, none of the included meta-analysis reported any adverse events in patients receiving these supplements, which seems to be safe for PCOS patients. Inconsistent results reported in different meta-analyses may be due to the differences in methodology, intervention duration, baseline characteristics or ethnicity of participants, and variation in probiotic strains and doses. Evidence shows that different strains and doses of probiotics exert different effects. Moreover, a longer intervention duration might be more effective. The most used probiotics in included studies belonged to *Lactobacillus* and *Bifidobacterium* strains; however, due to small sample sizes of the RCTs and high variation in used strains, subgroup analysis based on different probiotic strains was not performed in included SRs. Only Li et al. carried out a pre-planned subgroup analysis based on the number of probiotic strains used in formulation (multiple strains or single strain) and probiotics dose (≥2 × 10^8^ colony-forming units (CFU) or <2 × 10^8^ CFU), but not different strains. The number of included RCTs in meta-analysis carried out by Li et al. was 17 (the most number of included studies among the 8 meta-analyses) and included RCTs evaluated various forms of probiotics/prebiotics/synbiotics (9 trials for probiotics, 2 trials for prebiotics, and 6 trials for synbiotics). They showed no significant changes in BMI and TC by probiotic or synbiotic supplementation, while subgroup analyses regarding the type of intervention and study duration indicated that BMI and other anthropometric indices were reduced in trials with prebiotic supplementation and study duration of <12 weeks. Furthermore, TC and CRP decreased significantly with prebiotic supplementation, study duration of ≥12 weeks, and probiotic dose of ≥2 × 10^8^ CFU (24). Hadi et al.'s meta-analysis also showed no significant effect of probiotic or synbiotic administration on BMI but indicated a reductive effect on ≥30-year-old participants. However, the low methodological quality of this meta-analysis must be taken into account in the interpretation of the results (22). Since evidence is still scarce, it remains difficult to identify optimal probiotic strains, prebiotic types, length of duration, and doses.

**Figure 2:**
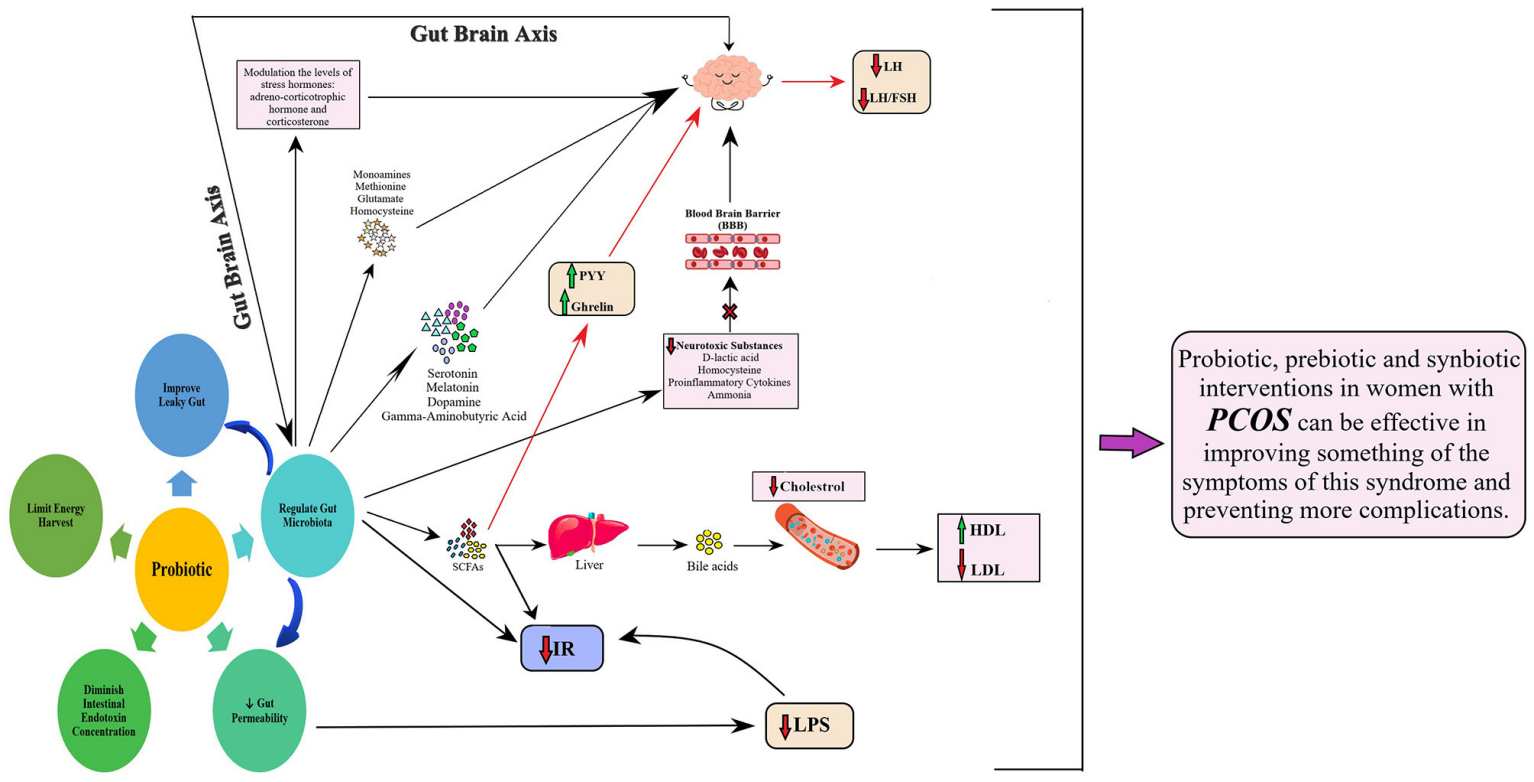
Underlying mechanism relating to the probiotic role in PCOS.

Probiotics/prebiotics/synbiotics might be a promising approach to improving body weight, insulin sensitivity, lipid profiles, and other PCOS-related parameters through different mechanisms (Figure 2). These products can regulate the gut microbial community's composition, improve leaky gut, decrease gut permeability, diminish intestinal endotoxin concentrations, and limit energy harvest (29–31). The gut microbiota–brain axis, particularly the hypothalamic signals, is also known to play an important role in regulating appetite, body weight, and whole-body metabolism (3, 32). The gut microbiota can affect the nervous system by producing and releasing some neuroactive compounds including serotonin, melatonin, dopamine, and gamma-aminobutyric acid. They also synthesize metabolites including monoamines, methionine, glutamate, and homocysteine, which can influence nervous system activity and modulate behaviors (33). A possible mechanism justifying the role of gut microbiota in brain functions is modulating the levels of stress hormones, such as adrenocorticotropic hormone and corticosterone, by gut microbiota. The gut microbiota creates neurotoxic substances, such as D-lactic acid, homocysteine, pro-inflammatory cytokines, and ammonia, which can pass the blood–brain barrier (BBB), reach the brain, and consequently affect the gut–microbiota–brain axis by the immune, neuroendocrine, and direct nervous mechanisms (34–36).

Probiotics/prebiotics/synbiotics could influence lipid profiles by improving the gut microbiota composition, enhancing the excretion of cholesterol by feces, modulating the metabolism of bile acids, and increasing the production of short-chain fatty acids by selective fermentation (37–39). Studies have demonstrated that probiotics have positive effects on inflammation and oxidative stress. Intake of probiotics or synbiotics seems to reduce inflammatory cytokines, lipid peroxidation, generation of hydrogen peroxide radicals, and oxidative damage via producing short-chain fatty acids in the intestine (40). The most used probiotics in humans belong to *Lactobacillus, Bacillus*, and *Bifidobacterium*, but also *Saccharomyces* is widely adopted in commercial products. Specific strains of *Lactobacillus* may modulate cytokine production by immune cells, and *Bifidobacterium* spp. could induce tolerance acquisition (41). Such different regulatory activities by each probiotic strain are linked to their structure, the spectrum of mediators released, and various pathways that are simultaneously activated (42). Nonetheless, specific molecular interactions between probiotics and host cells are not well-defined, and more research studies are needed in this regard.

Some studies have reported the effectiveness of probiotics/prebiotics/synbiotics in improving hormonal indicators in patients with PCOS. The results of one meta-analysis showed that the consumption of probiotics/prebiotics/synbiotics significantly reduced FAI and SHBG levels but did not significantly reduce testosterone levels (26), while another meta-analysis reported a significant reduction in total testosterone by both probiotic and synbiotic administration (28). The uptake of probiotics/prebiotics/synbiotics can improve hormonal status in PCOS by different mechanisms including regulating the colony of intestinal microbes and intestinal pH, improving intestinal digestion and absorption of nutrients, and affecting the production of inflammatory cytokines. These products also decrease cholesterol levels by reducing its production in the liver and decrease blood glucose and insulin resistance by consuming the serum insulin, which, in turn, reduced the production of androgens, such as testosterone, FAI, DHEAS, and SHBG levels (43, 44). However, as mentioned previously, few studies measured the hormones as the outcome, and increasing evidence is needed in this regard.

Probiotics/prebiotics/synbiotics have been approved for reducing PCOS symptoms via modulating gut microbiota, increasing proportions of *Bifidobacterium* and *Lactobacillus*, restoring the microbiota balance, reducing intestinal permeability, and decreasing translocation of lipopolysaccharides from the intestine to the blood circulation (45).

In our overview, we systematically searched and summarized all systematic reviews investigating the effects of probiotics, prebiotics, and synbiotics on polycystic ovarian syndrome. Moreover, the quality assessment of the meta-analyses has been conducted using the well-established AMSTAR-2 appraisal tool to enable a critical appraisal of the included systematic reviews. Although the overview of systematic reviews provides a broad perspective on interventions and their relative effectiveness, it inevitably has some limitations. First, the differences among studies regarding the type of intervention, study duration, strain numbers, probiotic dose, and other factors lead to statistical heterogeneity. Differences in participants' characteristics, such as age, gender, and ethnicity, make interpreting data and determining the best strain and dose of probiotics impossible. Moreover, many systematic reviews did not perform subgroup analyses and meta-regression because of a limited number of eligible studies or lack of data. Further studies are still needed in this regard.

## 5. Conclusion

The evidence from this study suggests that probiotic/prebiotic/synbiotic supplements have beneficial effects on improving some PCOS-related parameters including BMI, FPG, and lipid profile. However, we are still far from providing guidelines for its clinical application because of the complex nature of the gut microbiota. In addition, due to limited evidence and high heterogeneity of the studies, it remains difficult to identify optimal probiotic strains, prebiotic types, length of duration, and doses. Future clinical trials and meta-analyses with higher quality are recommended to clarify the efficacy of probiotics/prebiotics/synbiotics on the management of PCOS and provide more accurate evidence.

## Data availability statement

The original contributions presented in the study are included in the article/supplementary material, further inquiries can be directed to the corresponding authors.

## Author contributions

This study was designed and data collection or processing was done by H-SE and SH-R. Analysis or interpretation was performed by H-SE and PA. Literature search was done by H-SE, MT, and FE. This study was written by PA, BM, and H-SE. All authors have read and approved the manuscript.
